# The economic consequences of local gas leaks with evidence from Massachusetts housing market

**DOI:** 10.1016/j.isci.2024.111483

**Published:** 2024-12-17

**Authors:** Xingchi Shen, Morgan R. Edwards, Yueming (Lucy) Qiu, Pengfei Liu

**Affiliations:** 1 School of International and Public Affairs, Shanghai Jiao Tong University, Shanghai 200030, China; 2 La Follette School of Public Affairs, University of Wisconsin—Madison, Madison, WI 53706, USA; 3 Nelson Institute Center for Sustainability and the Global Environment, University of Wisconsin—Madison, Madison, WI 53706, USA; 4 School of Public Policy, University of Maryland, College Park, MD 20742, USA; 5 Department of Environmental and Natural Resource Economics, University of Rhode Island, Kingston, RI 02881, USA

**Keywords:** Classification Description, Environmental policy, Social sciences, Economics

## Abstract

This study provides the first empirical evidence of the impact of leaks in natural gas distribution pipelines on nearby home prices. Using high-resolution property transaction data in Massachusetts and a difference-in-differences approach, we find that gas leaks significantly reduce nearby home prices by 2.61% ($11,700) on average. Damage to local greenery likely plays an important role in the negative impact of gas leaks on nearby home prices. The impact is economically and statistically significant for houses with high surrounding tree cover and not significant for houses with low surrounding tree cover. However, we fail to detect a significant difference in the impact before and after an information shock of a deadly natural gas accident. Our estimated benefits of fixing gas leaks are larger than the average costs per repair reported by utilities in Massachusetts, which supports actions by policymakers and environmental organizations to reduce methane emissions from gas distribution pipelines.

## Highlights

• Estimation of the impact of local natural gas leaks on home prices
• Nearby home prices decrease due to gas leaks
• Benefits of repairing gas leaks exceed associated costs
• Damage to local greenery plays a significant role

## Graphical abstract

**Figure:**
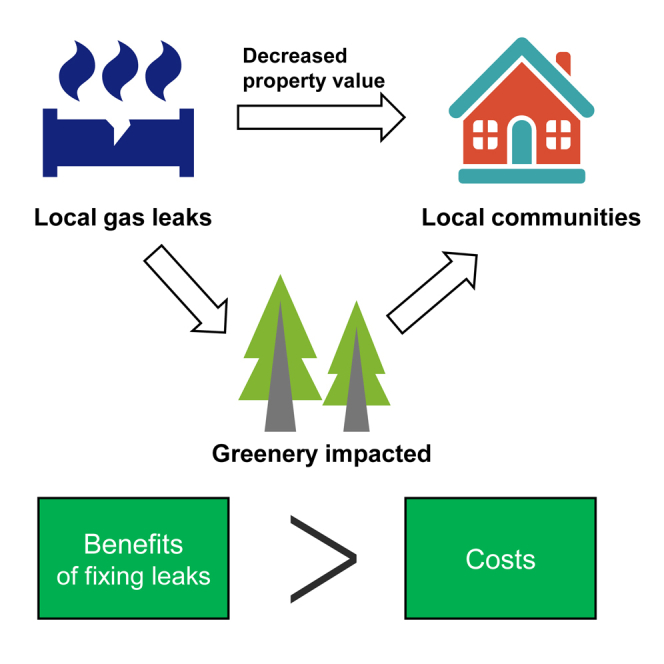

## Introduction

Reducing methane emissions is a key near-term policy lever to slow rates of warming while pursuing CO_2_ reductions through low-carbon electricity and decarbonizing energy end uses via electrification and energy efficiency.^1^^,^^2^ Methane is the second largest contributor to climate change after carbon dioxide (CO_2_).^3^ Compared to CO_2_, it is more efficient at trapping heat and has a shorter atmospheric lifetime of around a decade.^4^ Natural gas is primarily methane, and emissions throughout the supply chain contribute significantly to the climate impacts of natural gas systems, particularly on decadal time scales.^5^ Academics and policymakers are increasingly focused on reducing methane emissions from the natural gas system. National policy is primarily focused on upstream emissions, but state and local policymakers are increasingly interested in addressing downstream sources—including local distribution systems and, more recently, devices such as gas stoves and dryers that burn gas in buildings. Emissions from local distribution and end-uses can be critical for meeting state and local climate policy targets. Providing local residents and policymakers with significant economic incentives to fix these local gas leaks is essential for tackling global climate change.

In areas with aging natural gas distribution pipelines, including major cities along the U.S. East Coast, gas leaks can be a particularly important source of greenhouse gas emissions. For example, a previous study^6^ finds the combined yearly gas leak emissions from six U.S. East Coast cities are larger than those from the largest U.S. natural gas producers, such as the Bakken Shale in the Dakotas. In Massachusetts (MA), the location of our research case study, gas leaks account for approximately 10% of greenhouse gas emissions and are much higher than estimates in emissions inventories.^7^^,^^8^ Concerns about gas leaks prompted MA to require utilities to report leak and repair activity publicly beginning in 2014.^5^ By 2019, utilities had reported more than 80,000 gas leaks. Many leaks had not been fixed for decades, and new leaks continued to emerge, resulting in no decline in unrepaired leak counts and total emissions from the gas system in MA from 2014 to 2019.^8^

Local gas leaks present many problems beyond climate impacts. First, leaks can potentially pose an explosion risk. Gas explosion accidents in the U.S. can be related to gas leaks as well as other safety issues (e.g., line strikes). Gas explosions have caused casualties, destroyed houses, evacuated residents, and raised awareness of the potential impacts of using natural gas for local direct energy needs. Second, methane kills surrounding trees and plants by suffocating them at their roots, which damages local greenery.^9^ Local greenery offers multiple benefits such as enhancing views, providing shade, and increasing privacy, and thus can influence home prices.^10^ Third, gas leaks increase customers’ energy bills because utilities may pass the cost of lost gas to rate payers. Fourth, leaked gas is a waste of resources. Replacing gas lost through leaks requires more gas to be extracted, which leads to various social and environmental impacts on nearby communities, including those associated with hydraulic fracturing as well as transmission pipelines.^11^^,^^12^^,^^13^

**Figure 1:**
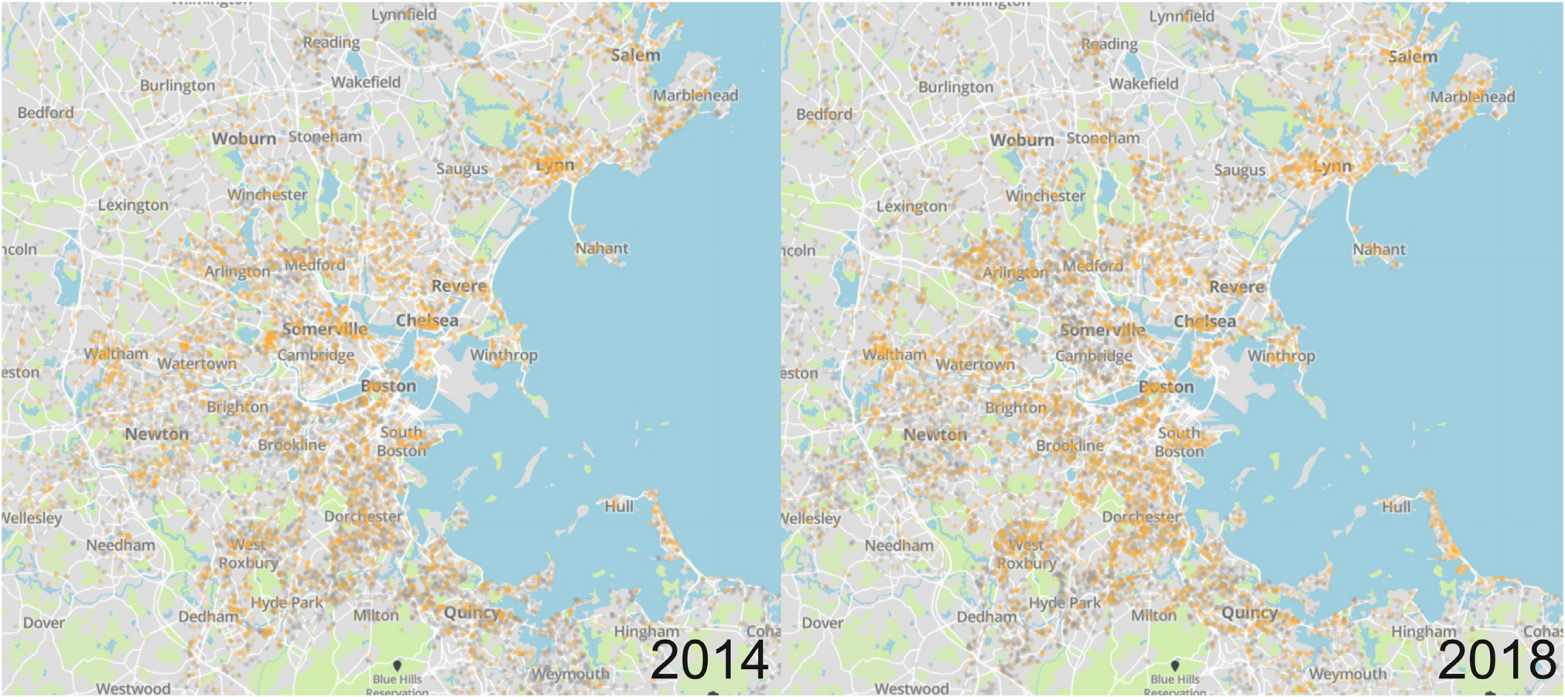
Repaired (gray) and unrepaired (orange) leaks reported by the three largest gas utilities in MA in 2014 and 2018 Source: Massachusetts Department of Public Utilities File Room.

Figure 1 shows the distribution of repaired and unrepaired gas leaks in 2014 and 2018 in MA. In 2014, the three major utilities reported 12,588 repaired leaks and 16,941 unrepaired leaks, while in 2018, they reported 10,134 repaired and 15,979 unrepaired leaks. Although the total number of reported leaks—both repaired and unrepaired—decreased slightly from the first to the last period, there remains a significant backlog of unrepaired gas leaks. The main obstacles faced by gas utilities include high repair costs—and difficulty raising funds to cover these costs -- as well as high rates of repair failures.^5^ Repairing leaks can also involve blocking local transportation, interrupting local gas supply, digging up the road, and restoring natural and street landscapes, which incur high private and social costs. Social planners need to evaluate if and when repairs are warranted by comparing the benefits with the associated costs.^14^ While the costs of repairing gas leaks are relatively easy to quantify, the benefits (i.e., the value communities place on repairs) are poorly understood.

This study estimates the impact of leaks in natural gas distribution pipelines on nearby home values in MA using high-resolution spatial data and a repeat-sales model. Surrounding features of a property, including the presence of gas leaks, can be reflected in housing prices based on a hedonic model framework.^15^ Since not every homebuyer notices a gas leak, homebuyers’ willingness to pay (WTP) to avoid gas leaks would likely be underestimated, and the estimated impact can be interpreted as the lower bound of the average home buyer’s WTP. Homebuyers who do not notice a gas leak directly may still pay less for the home due to other effects of these leaks, such as damage to surrounding greenery. Our results suggest that local gas leaks (including all grades, where grade is a measure of safety risk) significantly reduce nearby home prices by 2.61% ($11,700) on average in MA. Grade 2 leaks, which are considered a probable future safety risk, significantly reduce the home prices by about 11% ($53,800) on average, while grade 3 leaks (considered not a safety risk but potentially a large emissions source) decrease the home prices by 1.97% ($8,800). Our estimations are robust to different treatment group buffer lengths and control group buffer lengths.

We also provide empirical evidence on two potential channels behind the negative impact of gas leaks on nearby home prices. First, leveraging high-resolution tree cover data derived from satellite images (30 m per pixel), gas leaks decrease nearby home prices significantly for houses with high surrounding tree cover but do not have a significant impact on houses with low surrounding tree cover (controlling for household income). This result is likely due to the fact that gas leaks can cause damage to local greenery, which contributes to overall home value.^10^ Second, we test whether the impact of leaks on home prices changes after the exogenous information shock of the Merrimack Valley gas explosion accident. While the accident was due to operator error and not a gas leak, the deadly accident and following wide media coverage raised awareness about natural gas system risks. However, we find that the impact on home prices did not change significantly following the accident. Some homebuyers may not notice the gas leak even if their risk perception of natural gas explosion changes.

Our study underscores the economic and policy benefits of reducing gas leaks, particularly in areas with aging infrastructure such as MA. The findings support policy discussions and provide valuable insights for environmental organizations, homeowners, and cities with similar challenges, such as Washington, DC, Philadelphia, PA, and others.^6^^,^^16^

## Results

### The impact of gas leaks on nearby home prices

We combine high-resolution spatial data on gas leaks, which includes the address of the leak and dates each leak was discovered and repaired, with property transaction records to create a repeated-sales sample of homes located near gas leaks. Using the difference-in-differences (DID) method, we estimate the impact of gas leaks on nearby house sales prices. The estimated impact can be interpreted as the average homebuying population’s lower bound of WTP for avoiding gas leaks. When individuals are assumed to choose the living location to maximize utility, the surrounding features of a residential property, including nearby environmental qualities or risks such as gas leaks, will be reflected in the house sales prices. This approach, namely the hedonic pricing model, has been widely used^15^^,^^17^^,^^18^^,^^19^^,^^20^^,^^21^ to estimate the value of environmental (dis) amenities. If gas leaks are found to have a negative impact on home prices, the societal WTP for avoiding the leak is positive.

When there is a gas leak close to a house, homebuyers could smell the natural gas because mercaptan is added to the natural gas by utilities. Mercaptan smells such as sulfur, or rotten eggs, and helps residents identify gas leaks easily. Even though some consumers may not smell the gas, they could notice the dead trees or other plants killed by nearby gas leaks. Moreover, an online map showing all the locations of reported gas leaks in MA was published by HEET (Home Energy Efficiency Team, heet.org) in 2015 and is updated annually. Residents can search for the location of a gas leak in MA. Homebuyers who care more and have a higher WTP may be more likely to check the online gas leak map and notice a nearby gas leak, whereas those who do not may have a lower but nonzero WTP. We thus interpret our estimation of the impact on home prices as the lower bound of the average population’s WTP since we may underestimate the WTP of those who did not notice the gas leaks around the time of sale.

**Figure 2:**
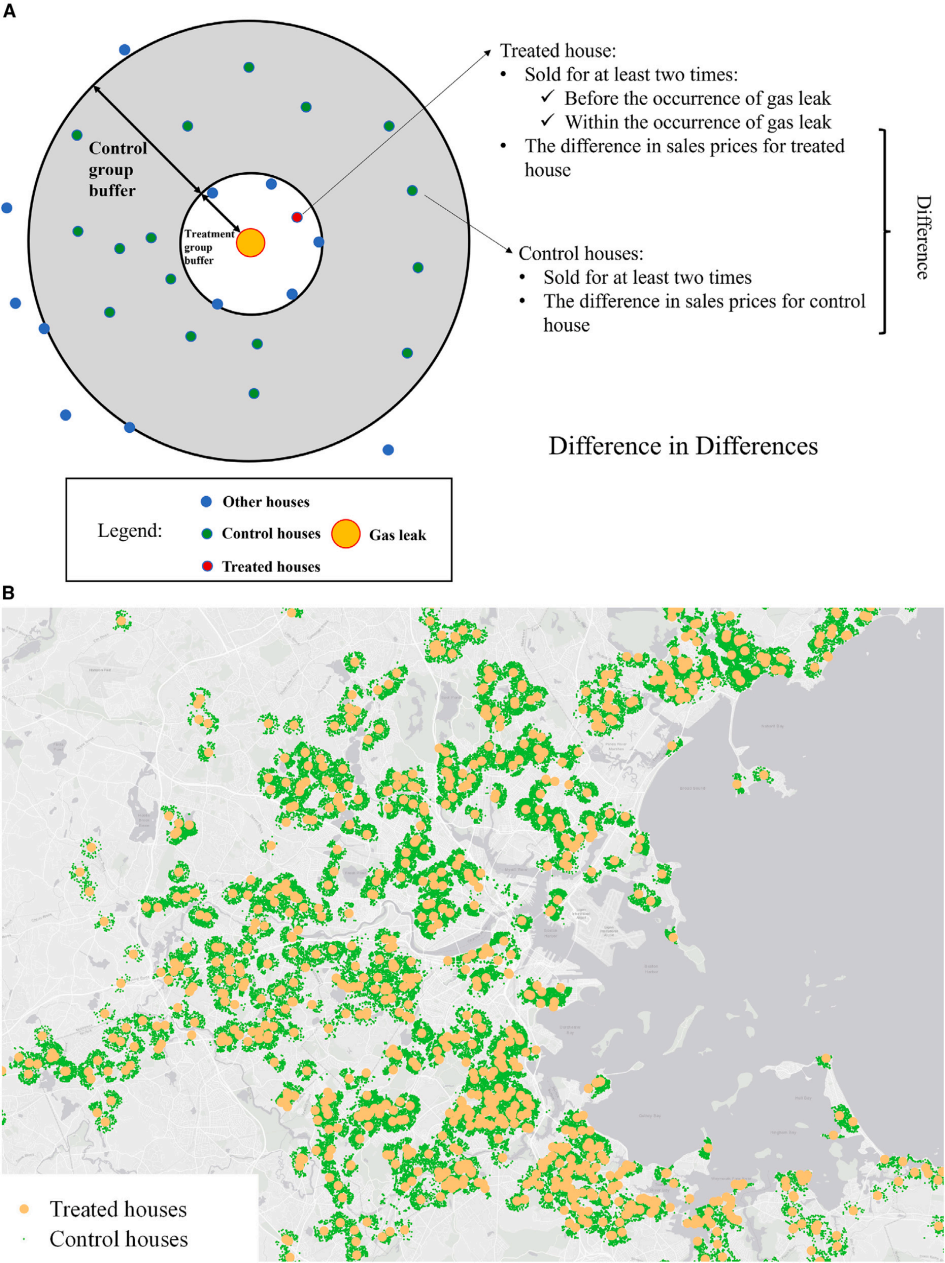
Illustration of methods (A) method for identifying control and treated houses. (B) distribution of selected treated and control houses.

In our DID design, the treatment group consists of houses with a nearby gas leak. The lot size of a typical house in areas with gas leaks in MA is about 1,200 square meters, which equals the area of a circle with a radius of 20 m. Thus, in our baseline analysis, we define the “nearby gas leaks” as leaks within 20 m of houses and assume that home prices can only be influenced by nearby gas leaks. We validate this assumption in Data S1. For each leak, we apply a matching algorithm to find all the residential buildings within 20 m of the leak. The house closest to the leak enters the treatment group. In addition, houses within 20 m–500 m of the leak are chosen to be the control group. To avoid the overlap of treated and control houses, we excluded all houses with nearby gas leaks from the control group. The treated houses were sold at least once before the occurrence of a gas leak and once within the occurrence of a gas leak. The control houses were also sold at least twice during a similar time window. Under this DID framework, we selected 1,350 houses in the treated group and 188,640 houses in the control group. See Figure 2 for an illustration of the identification and distribution of selected treated and control houses. Thanks to the high geographical resolution data, we are able to construct local comparable groups to control for the influence of location on home prices.

We further compare home characteristics between the two groups (see Table 1). The treated and control houses are similar in terms of the magnitude of most observed building features, although many *p*-values are less than 0.1 based on t-tests, potentially because of the large sample size. Nonetheless, the identification of DID models does not require perfect balance in levels of attributes between the treated and control groups as long as time-invariant house attributes can be differenced out. To show the plausibility of the parallel trend assumption, we conduct and pass a pre-treatment parallel trend test (see STAR Methods and Figure S1). To investigate the sample selection issue, we compare the selected treated group with all other houses in MA (see Table 1). Most observed building features (e.g., year remodeled; number of stories, rooms, bedrooms, and bathrooms; building area) are similar between selected homes and other homes. However, we find that the treated buildings are older, smaller (in terms of lot size), more expensive (in terms of land value), more likely to have a garage and a fireplace, and less likely to have a swimming pool than all other houses in MA. This may be because gas leaks are more likely to occur in an urban area than in a rural area, and treated houses are thus more likely to be distributed in the urban area.

**Table 1:** Descriptive statistics of selected sample and all other houses in MA

	Treated houses (*n* = 1,350)	Control Houses (*n* = 188,640)	All other houses in MA (*n* = 2,208,362)	P-value (1) versus (2)	P-value (1) versus (3)
	*(1)*	*(2)*	*(3)*	*(4)*	*(5)*
**Mean House Characteristics**					

Year Built	1932.10	1935.06	1950.82	0.01	<0.01
Year Remodeled	1984.48	1987.34	1985.05	0.02	0.6
Lot Size (Acres)	0.34	0.32	1.20	0.67	<0.01
Lot Size (Sq Ft)	14760.79	13878.44	52156.25	0.67	<0.01
No of Stories	1.99	1.86	1.78	<0.01	<0.01
Total Rooms	8.01	7.41	7.42	<0.01	<0.01
Total Bedrooms	3.66	3.36	3.43	<0.01	<0.01
Total Calculated Bath Count	2.14	1.98	2.05	<0.01	<0.01
Building Area (SqFt)	2117.35	1894.47	2079.49	<0.01	0.68
Garage (Dummy)	0.39	0.39	0.29	0.77	<0.01
Pool (Dummy)	0.03	0.03	0.04	0.98	0.01
Land Assessed Value ($)	286535.80	243883.60	244047.00	0.05	0.19
Fireplace (Dummy)	0.41	0.32	0.36	<0.01	<0.01

The sample of columns (1) and (2) (treated and control houses) includes all the houses in our baseline DID sample. The sample of columns (1) and (3) (treated and all other houses in MA) includes all the houses in MA. Column (4) reports *p*-values from tests that the means are equal in the two subsamples (1) and (2). Column (5) reports *p*-values from tests that the means are equal in the two subsamples (1) and (3).

We then apply a two-way fixed-effects model to estimate treatment effects by regressing the log of home sales prices on a treatment variable, which takes a value of one for treated homes in the post-treatment period, controlling for building age, individual household fixed effects, year fixed effects, and month-of-year fixed effects. We cluster the standard error at the individual household level. Table 2 shows baseline estimation results by leak grade: grade 1 leaks are hazardous and must be repaired immediately; grade 2 leaks are potentially hazardous and required to be repaired within one year; and grade 3 leaks are non-hazardous and not required to be repaired but must be re-evaluated at regular intervals. Column (1) shows the estimation of the impact of gas leaks at all grades. We find that the gas leaks reduce the nearby home prices by 2.61%, or $11,700, on average (*p*-value = 0.1). Column (2) excludes grade 1 leaks from the sample. The result is not very sensitive to this change (3.29% reduction on average, *p*-value = 0.04) as grade 1 leaks account for a small proportion of our sample. Columns (3) and (4) investigate the impact of grade 2 leaks and grade 3 leaks separately. We find that grade 2 leaks significantly reduce home prices by about 11% (*p*-value = 0.06). Grade 3 leaks only decrease prices by 1.97%, and this result is not statistically significant (*p*-value = 0.22). One factor determining leak grade is how close a leak is to a building, with closer leaks are more likely to be grade 2. These leaks may be more likely to be detected by homebuyers and thus explain the greater impact of grade 2 leaks.

**Table 2:** The impact of gas leaks on nearby home prices

	(1)	(2)	(3)	(4)
	Outcome: Natural log of home prices (2019$)			
	*All grades*	*Grades 2 and 3*	*Grade 2*	*Grade 3*
Gas leak	−0.0261∗ (0.0159)	−0.0329∗∗ (0.0157)	−0.1138∗ (0.0602)	−0.0197 (0.0162)
Building age control	Yes	Yes	Yes	Yes
Individual FE	Yes	Yes	Yes	Yes
Year FE	Yes	Yes	Yes	Yes
Month FE	Yes	Yes	Yes	Yes
Adjusted R^2^	0.26	0.26	0.29	0.26
Observations	547760	540741	69491	471250

Standard errors are in the parentheses and clustered at the individual household level. ∗∗∗*p* < 0.01, ∗∗*p* < 0.05, ∗*p* < 0.1. The outcome is the natural log of home prices that are adjusted to 2019$ for inflation. During the post-treatment period, the treated buildings were traded with a nearby gas leak. Transactions with the treatment (gas leak) happened from 2015 to 2019.

Different utilities may have different repair costs, and people living in their service territories may also have different WTPs for avoiding gas leaks. We further estimate the impacts of gas leaks on home prices by utility service territory for the three largest utilities (National Grid, Columbia Gas, and Eversource) in MA at the time of the study. For each utility, we report outcomes by leak grade (all grades, grade 2, and grade 3) in Table 3. We do not estimate the impact of grade 1 leaks due to the small sample size. Two estimates in columns (1) and (3) are statistically significant: all grades and grade 3 leaks in the National Grid service territory, which includes the Boston metropolitan area, both significantly reduce the nearby home prices by 4%. The gas leaks in the other two utilities do not have a significant impact on nearby home prices. We also compare the building attributes of treaded houses and local demographic characteristics across utility service territories (see Table S1). We find that the treated houses in the National Grid territory are older and more densely distributed with smaller lot sizes but with higher surrounding tree coverage. Residents also have higher education and income levels compared to the other two utility areas. Thus, residents in the National Grid utility area may be more likely to notice the gas leak and be willing to pay more to avoid it, which contributes to the significant impact on home prices.

**Table 3:** The impact of gas leaks on nearby home prices by utility

	(1)	(2)	(3)	(4)	(5)	(6)	(7)	(8)	(9)
	Outcome: Natural log of home prices (2019$)								
	National Grid			Columbia Gas			Eversource		
	All grades	Grade 2	Grade 3	All grades	Grade 2	Grade 3	All grades	Grade 2	Grade 3
Gas leak	−0.04∗∗ (0.02)	−0.06 (0.07)	−0.04∗ (0.02)	−0.04 (0.04)	−0.16 (0.12)	−0.04 (0.04)	0.04 (0.03)	−0.05 (0.07)	0.04 (0.03)
Building age control	Yes	Yes	Yes	Yes	Yes	Yes	Yes	Yes	Yes
Individual FE	Yes	Yes	Yes	Yes	Yes	Yes	Yes	Yes	Yes
Year FE	Yes	Yes	Yes	Yes	Yes	Yes	Yes	Yes	Yes
Month FE	Yes	Yes	Yes	Yes	Yes	Yes	Yes	Yes	Yes
Adjusted R^2^	0.29	0.33	0.29	0.26	0.3	0.26	0.22	0.18	0.22
Observations	330398	49367	279202	101549	16324	80754	115813	3800	111294

Standard errors are in the parentheses and clustered at the individual household level. ∗∗∗*p* < 0.01, ∗∗*p* < 0.05, ∗*p* < 0.1. The outcome is the natural log of home price adjusted to 2019$ for inflation. During the post-treatment period, the treated buildings were traded with a nearby gas leak. Transactions with the treatment happened from 2015 to 2019. The three largest utilities in MA were National Grid, Columbia Gas, and Eversource in period of our study.

Under the baseline specification, we assume that the prices of houses that are 20 m away from the leak are not influenced by the leak, since it is hard for people to detect the gas at that distance. One concern is that buyers of houses 20 m or more from a leak may still notice dead plants caused by the leak and could find the leak location from the online gas leak map, and thus their home buying decision may be influenced by the leak. To relieve this concern and test our assumption about the control group buffer, we conduct a robustness check by expanding the internal boundary of the control group and holding the boundary of the treatment group fixed. We find that estimations are not sensitive to the changing control group buffer, which indicates that gas leaks do not influence home prices outside a radius of 20 m (see details in Data S1). Another concern is that our estimated treatment effect may change if we change the radius of the treatment group buffer, since some home prices within our defined treatment group boundary may not be influenced by the gas leaks. To relieve the concern about the treatment group buffer, we conducted another robustness check by shrinking the boundary of the treatment group and holding the control group buffer fixed. We find consistent results (see details in Data S1).

### Understanding the impact of gas leaks on home prices

To understand the estimated impact of local gas leaks on home values, we explore two possible mechanisms. Local gas leaks impose four types of costs: methane emissions (and associated climate and air quality impacts), increased gas bills, tree and plant deaths, and potential explosions. The first two channels are unlikely to significantly affect home prices since the impacts of methane emissions are global, and an outside gas leak will not directly increase the gas meter readings of a nearby household. Even though gas utilities may pass the cost of leaking gas to all consumers, this cost does not depend on the location of the leak within the service territory. Thus, the negative impact of gas leaks on home prices could be explained by two potential mechanisms.

(1) Damage to local greenery. A gas leak can kill trees and other plants around a house, and homebuyers will be willing to pay less for a house with poor greenery.
(2) Potential explosions. If a homebuyer notices the gas leak and thinks it has the potential to explode, they will be less willing to purchase the house.

In the following sections, we explore these two potential influence channels.

#### Damage to local greenery

Leaked gas kills surrounding plants by suffocating them at their roots, which damages surrounding greenery and decreases home values. To explore this potential influence channel, we obtained satellite tree cover data for 2019 (30 m by 30 m resolution) to measure local greenery around the buildings in our sample. This high-resolution geographical data enables us to identify the density of greenery near each building based on latitudes and longitudes. A 30 m by 30 m cell could be too small to reflect the surrounding greenery because a house’s floor space can be as large as 30 m by 30 m. Thus, we identify 9 cells around each building (the cell containing the building’s longitude and latitude and its 8 neighboring cells). We compute an average of tree cover across the 9 cells to represent the local greenery around each building. Figure S2 plots the distribution of tree cover around buildings in our sample. The median tree cover in the sample is 18.1%.

To illustrate whether damage to local greenery can influence WTP to avoid gas leaks, we estimate the impact of gas leaks on home prices for different levels of tree cover. We divide the tree cover into high tree cover and low tree cover according to the median value and create two dummy variables to indicate these two categories. Using the same DID model in our baseline specification, we interact the treatment variable with the two indicator variables. The two categories of tree cover could be endogenous in our model since homes with high tree cover tend to have better views and be located in high-income areas. Wealthier households tend to have a higher WTP for environmental amenities and health benefits,^14^ which may confound the estimates of the interaction terms in our model. Thus, we include yearly median household income (from 1989 to 2019) at the county level (We are unable to control the income at a smaller level, such as the census tract level, for two reasons: (1) The income data at census tract level is only available from 2010 to 2020 from the U.S. Census Bureau. If we use it, we need to restrict our time window and cut a lot of data. (2) The income data at the tract level is only available from ACS 5-year estimates. The ACS 5-year estimates are based on observations across 5 years. There is no 1-year estimate at the tract level. Since it is important to leverage the time-series variation in our model, 5-year estimates are not suitable for our model.) in the model to control for potential confounders.

Table 4 presents estimates of the effects of gas leaks for homes with high and low tree cover. Column (1) uses the whole sample and shows that the impact of gas leaks on home prices in communities with low tree cover is not significant, while the impact in communities with high tree cover is negative with both statistical and economic significance. A gas leak can decrease home price by 3.99% for communities with high tree cover. Column (2) uses the sample of houses with grade 3 leaks and finds consistent results. We also conducted F tests to compare the difference between the coefficients of two interaction terms. The coefficients are significantly different for houses with all grades of leaks in column (1). We did not use the sample of houses with grade 2 leaks for estimation since the number of grade 2 leaks in our sample is limited for high greenery houses. Our results provide evidence that local greenery plays an important role in the impact of gas leaks on home prices. Damage to local greenery is larger and more noticeable by homebuyers in communities with high tree cover, and homebuyers are willing to pay less for a property with the lower cover of surrounding greenery (We provide additional empirical evidence that diminished greenery reduces home prices in Data S2.). Additionally, damaged greenery may make homebuyers more likely to detect gas leaks and perceive additional concerns related to leaks beyond just tree death.

**Table 4:** The role of tree cover and risk perception on gas leak impacts on home prices

	(1)	(2)	(3)	(4)
	Outcome: Natural log of home prices (2019$)			
	All Grades	Grade 3	All Grades	Grade 3
Gas leak ∗ Low tree cover (a)	0.0218 (0.0327)	0.0224 (0.0328)		
Gas leak ∗ High tree cover (b)	**−0.0399∗∗ (0.0188)**	**−0.0355∗ (0.0190)**		
Gas leak			−0.0755 (0.0648)	−0.0467 (0.0536)
Gas leak ∗ post-explosion			0.0172 (0.0836)	0.0051 (0.078)
Building age control	Yes	Yes	Yes	Yes
County yearly median household income	Yes	Yes	Yes	Yes
Individual FE	Yes	Yes	Yes	Yes
Year FE	Yes	Yes	Yes	Yes
Month FE	Yes	Yes	Yes	Yes
Adjusted R^2^	0.1687	0.1687	0.26	0.26
Observations	460,212	395,730	117,938	103,758
F-statistic of (a) versus (b)	2.70	2.35	–	–
P-value of (a) versus (b)	0.1006	0.1249	–	–

Standard errors are in the parentheses and clustered at the individual household level. ∗∗∗*p* < 0.01, ∗∗*p* < 0.05, ∗*p* < 0.1. The outcome is the natural log of home prices that are adjusted to 2019$ for inflation. For columns (1) and (2), we interact the treatment variable with two indicators of high tree cover and low tree cover, respectively. For each regression, we conduct an F-test to compare the difference between the coefficients of two interaction terms. For columns (3) and (4), the interaction term is between the treatment variable and an indicator variable which takes value one for dates after September 13, 2018.

#### Knowledge and risk perception

The second source of the negative impact of gas leaks on home prices could be a homebuyer’s perception of the risks of potential explosions. When homebuyers notice a nearby gas leak, they may worry that the leak will explode and endanger their safety, which will decrease their WTP for the house. Knowledge and risk perceptions about potential explosions could be a key parameter in determining the WTP for avoiding the gas leak. The unexpected, deadly Merrimack Valley gas explosions in MA on September 13, 2018, enable us to explore the role of risk perception in the impacts of gas leaks on home prices. In 2018, a human error in the pipeline repair caused excessive pressure in gas pipes and led to a series of explosions and fires covering over 40 homes in the towns of Lawrence, Andover, and North Andover, MA. One person was killed, and 30,000 residents were forced to evacuate homes immediately. A large amount of media coverage followed, and this exogenous information shock could increase residents’ perceived risk of gas leaks and WTP for avoiding the leaks.

To test our hypothesis, we compare the impacts of gas leaks on home prices 3 months before and after the Merrimack Valley explosions. This comparison within a short period helps minimize confounding influence of unobserved time-series trends. Using the same DID model in our baseline estimation, we add an interaction term between the treatment variable and an indicator variable which takes a value one for dates after September 13, 2018. The coefficient of the interaction term measures the difference in the impact of gas leaks on home prices before and after the accident. Time-series variation in factors that are unrelated to the information shock of the accident may confound our comparison. For instance, economic growth in MA may lead to an increase in household income and thus influence WTP for environmental amenities (e.g., local greenery). Wage growth is also related to risk pref. ^22^ and the value of statistical life.^23^ To address this concern, we control for yearly median household income at the county level. Local environmental organizations (e.g., HEET) also organized many waves of public campaigns to raise MA residents’ awareness of gas leaks. Most campaigns were conducted from 2016 to 2018, with two campaigns in Weston and Needham in 2019. To relieve the confounding impact of public campaigns, we remove the observations in Weston and Needham from our sample. Additionally, we exclude observations from towns where the explosion occurred (e.g., Lawrence, Andover, and North Andover) in the post-explosion period. This approach eliminates the impact of the explosion on the housing market other than information shock, such as potential damage or other influences on the local building stock. Figure S3 shows the spatial distribution of treated homes used in this analysis.

Columns (3) and (4) in Table 4 present the estimation results for the effects of the Merrimack Valley information shock. We find that there is no significant difference before and after the accident using the whole sample or houses with grade 3 leaks. Consumers’ insignificant response could be due to the fact that some homebuyers did not notice the gas leak when buying homes. Although consumers’ knowledge and perceived risk of potential explosions is a key parameter in determining WTP for avoiding gas leaks, it may play a relatively small role in the impact of gas leaks on home prices when some gas leaks are hard to detect directly.

## Discussion

This study provides the first empirical evidence on the impact of local gas leaks on nearby home prices using high-resolution geographical data in property transactions and a DID method based on a repeat-sales sample. Our empirical evidence shows that, across all grades, gas leaks significantly reduced the nearby home prices by 2.61% ($11,700) on average in MA. Our estimates are robust to changing the boundaries of the control and treatment groups. We also find that gas leaks decreased home prices significantly in high-tree-cover communities but did not have a significant impact in low-tree-cover communities. There was no significant increase in the negative impact of gas leaks on home prices after an unexpected explosion accident with wide media coverage. These findings suggest that damage to local greenery may play an important role in explaining the impact of gas leaks on home prices, while consumers’ risk perception for potential explosions may play a relatively small role or may not be sensitive to media coverage of accidents that highlight the safety risks posed by the gas system.

We conduct a back-of-envelope analysis to compare the benefits and costs of fixing gas leaks. Since not every homebuyer will notice a gas leak, our estimates can be interpreted as the lower bound of individuals’ WTP for avoiding gas leaks, as well as a lower bound of benefits of fixing gas leaks that does not reflect additional societal benefits (e.g., reduced climate impacts). Whether fixing gas leaks is warranted is determined by comparing the costs with the benefits. Based on utility-reported data from the federal Pipeline and Hazardous Materials Safety Administration,^5^ a total of 17,533 unrepaired leaks were reported in MA at the end of 2018, with an estimated repair cost of $59,260,458. Thus, the average cost per repair is $3,380, which is much smaller than the benefits of repair. The repair costs reported by utilities also generally do not include the cost of restoring dead or damaged greenery after the repair. Recovery of greenery (especially larger trees) may take years. As an alternative estimate, we add the cost of tree planting to the cost reported by utilities. The typical cost of transplanting five medium trees (8′ to 10′ tall) on a residential lot is about $1,000, bringing the total cost to $4,380. Our estimated benefit of fixing the leaks is still much larger than the cost.

This study has broad implications for gas leak repairs and home heating and cooking transitions, especially in cities along the East Coast with aging natural gas infrastructure. MA is a state with significant community engagement and policy action on gas leaks. Recently, MA has passed multiple major pieces of legislation on gas system reporting, repair, and transition. Our results for MA provide evidence on the benefits of reducing gas leaks (through repair, replacement, or transition), which exceed the average cost of repair reported by utilities, contributing to active policy discussions in MA. Our findings can also be useful to other practitioners. Environmental organizations can use the estimated negative effect of gas leaks on home prices (consumers’ lower bound of WTP for avoiding the leaks) as references when lobbying legislators and carrying out public campaigns. For homeowners, our results encourage them to report the gas leaks as soon as possible to prevent economic losses. Our study has important implications for other locations with older natural gas distribution infrastructure where leak and repair data are not publicly available.

Public campaigns led by policymakers and local environmental organizations, along with the 2018 Merrimack Valley gas explosions, may have heightened public awareness of gas leaks in recent years. However, we find no significant increase in the negative impact of gas leaks on home prices after the information shock of a deadly explosion accident. This result could be due to the fact that some homebuyers in our sample may not notice gas leaks, or they do not associate the risk of operator error (the cause of the Merrimack Valley explosion) with the broader explosion risk of gas leaks. In addition, local environmental organizations have transitioned their campaigning strategies over time, from an initial focus on local risks and tree damage to global risks such as climate change.^5^ These concerns may further increase WTP to avoid gas leaks, which in turn would justify more investments in pipeline repair, replacement, and transition. Other activities that raise public awareness of gas leaks, including local campaigns that map and tag gas leaks, may have stronger impacts on local WTP to avoid leaks.

Our estimated effect on home prices can be interpreted as the average treatment effect on treated (ATT),^24^ while the benefits and costs of fixing gas leaks can vary across locations. One financial mechanism to cover the cost of fixing leaks is to uniformly raise gas rates, which poses a significant equity concern. In MA, one of the most racially segregated regions in the U.S., low-income households and households of color are more likely to live in older urban areas,^25^ due to historic discriminatory housing policies, such as redlining,^26^ as well as ongoing housing discrimination.^27^ In our study, we find that local greenery plays an important role in the impact of gas leaks on home prices. If greater benefits from fixing gas leaks accrue to homeowners in wealthier communities with more tree cover, while the costs are borne by everyone, marginalized populations will be disproportionately burdened. Another study also finds that marginalized populations are more likely to be exposed to local gas leaks.^28^ Future studies can explore the heterogeneity in benefits and costs of fixing gas leaks and approaches to equitable transitions in areas with leaky gas distribution infrastructure.^29^

### Limitations of the study

A limitation of this study is our limited understanding of awareness and perceptions of gas leaks, such as concerns about impacts on health or gas bills. While in-depth interviews or surveys are beyond the scope of this work, they are crucial for future research to gain deeper insights into gas leak perceptions.

## Resource availability

### Lead contact

Further information and requests for resources should be directed to and will be fulfilled by the lead contact, Yueming (Lucy) Qiu (yqiu16@umd.edu).

### Materials availability

This study did not generate new unique reagents.

### Data and code availability

• This study uses public data on gas leaks, tree cover, and census demographics (access links provided in key resources table); ZTRAX property data require a request to Zillow company.
• This article does not report the original code.
• Any additional information required to reanalyze the data reported in this article is available from the lead contact upon request.

## Acknowledgments

We thank Zeyneb Magavi, Chris Rea, and seminar participants at the Center for Global Sustainability, the Association of Environmental and Resource Economists summer conference, and the Association for Public Policy Analysis and Management fall research conference for helpful comments during the preparation of this article. Individual property data were provided by Zillow through the Zillow Transaction and Assessment Database (ZTRAX). More information on accessing the data can be found at https://www.zillow.com/research/ztrax/. The data are proprietary and are not publicly available under a non-disclosure agreement with Zillow. Interested readers can submit a request to Zillow for approval to obtain the data. This research did not receive any specific grant from funding agencies in the public, commercial, or not-for-profit sectors.

## Author contributions

X.S., M.R.E., Y.Q., and P.L. designed the study and planned the analysis. X.S. conducted the data analysis and drafted the article. M.R.E., Y.Q., and P.L. edited the article. All authors contributed to the interpretation of the findings.

## Declaration of interests

There is no competing interest.

## STAR★Methods

### Key resources table

**Table:** 

REAGENT or RESOURCE	SOURCE	IDENTIFIER
**Deposited data**		

Massachusetts gas leak data	Massachusetts Department of Public Utilities File Room	https://eeaonline.eea.state.ma.us/DPU/Fileroom
Zillow’s Assessor and Real Estate Database	Zillow company	From Zillow company directly
2010 Global Tree Cover data	Global Land Analysis and Discovery (GLAD) laboratory at the University of Maryland	https://glad.umd.edu/dataset/global-2010-tree-cover-30-m
2019 U.S. tree cover data	U.S. Department of Agriculture Forest Service	https://data.fs.usda.gov/geodata/rastergateway/treecanopycover/
Social demographic data in Massachusetts	U.S. Census	https://data.census.gov/

**Software and algorithms**		

STATA 15	This study	https://www.stata.com/stata15/
Python 3.8.8	This study	https://www.python.org/downloads/release/python-388/

### Method details

#### Data

We obtained utility-reported, state-wide data on gas leaks from the Massachusetts Department of Public Utilities File Room. Since 2014, MA law has required utilities to report these data to the public.^30^ Our dataset is collected from annual reports from 2014 to 2019. The combined gas leak dataset records approximately 80,000 gas leaks reported by six natural gas utilities in MA (including Berkshire Gas, Columbia Gas, Eversource Energy, Fitchburg Gas, Liberty Utilities, and National Grid). The data provides four important variables: the gas leak’s location (full address, which we used to map latitude, and longitude using the Google Maps API), leak grade (1, 2, and 3), date discovered, and date repaired. Gas leaks are divided into three grades. Because leak grade is an indicator of an explosion risk rather than the size of the leak, grade 3 gas leaks can also be large leaks. Many grade 3 gas leaks in our dataset have not been fixed for decades. The potential impacts of grade 2 and grade 3 leaks on home prices and the environment could be much higher than grade 1 because grade 1 leaks must repaired quickly, and thus their duration is very short.

We obtained data on building attributes and property transactions from the Zillow’s Assessor and Real Estate Database (ZTRAX) from the Zillow Group. The ZTRAX database has two parts: assessment and transaction data. Assessment data covers more than 150 million residential properties in over 3,100 counties and 50 U.S. states (and Washington, DC). We can observe detailed individual building features over ten periods from 03/22/2016 to 01/02/2020. These building assessments were conducted by county/town assessor offices for evaluating property taxes. The recorded building features include year built, year remolded, building quality, building condition, site characteristics, number of rooms/bathrooms/bedrooms, area, garage, and others. The transaction data has transaction records for each building since the early 1900s across the U.S. It records the sales price and date for each transaction. In this study, we only use the proportion of the dataset in MA, which includes 2,497,173 residential properties and 2,324,695 transaction records.

To support the mechanism analysis in our study, we obtained tree cover data from the U.S. Department of Agriculture Forest Service to measure local greenery around the buildings in our sample. The data records the estimates of maximum (at the peak of growth season) tree canopy cover percentage per geographical cell (30 m by 30 m) in 2019 in the U.S. based on satellite images. In our study, we only use the proportion of the dataset in MA, which includes 23,307,678 geographical cells (30 m by 30 m). We also obtained the yearly median household income at the MA county level from 1989 to 2019 from the American Community Surveys (ACS) of the U.S. Census Bureau.

#### Difference in differences

To mitigate potential selection and omitted variables bias^24^^,^^31^ in treatment effect estimation, we apply the DID method with matching based on the repeated sales sample. The treatment group consists of houses with a nearby gas leak. In our baseline analysis, we define the “nearby gas leaks” as leaks within 20 m of houses and assume that home prices can only be influenced by nearby gas leaks. Houses within 20 m–500 m of the leak are chosen to be the control group. The treated houses were sold at least once before the occurrence of a gas leak and once within the occurrence of a gas leak. The control houses were also sold at least twice during a similar time window of treated houses. A two-way fixed effects (generalized DID) model is applied to estimate the average treatment effect of gas leaks on home prices:

(Equation 1) $${\ln\;P}_{it}=\beta D_{it}+\alpha A_{it}+\phi_{i}+\vartheta_{t}+\tau_{t}+\varepsilon_{it}$$

where $\ln\;P_{it}$ is the log of the sales price of house *i* at time *t*. The price is converted into 2019 dollars adjusted for inflation rates. $D_{it}$ is the treatment variable, which takes value one when house *i* received the treatment (i.e., the occurrence of a gas leak) at the post-treatment period *t*. $A_{it}$ is the building age since it was built. $\phi_{i}$ represents the property fixed effects, capturing all time-invariant building-specific characteristics, such as architectural style, property address, building materials, and building area. $\vartheta_{t}$ is the year fixed effects, which captures unobservable common yearly trends (e.g., time-variant macro housing market conditions). $\tau_{t}$ is the month-of-year fixed effects, which controls variation over the annual cycle. $\varepsilon_{it}$ is an idiosyncratic error term. Standard errors are clustered at the individual building level, allowing for arbitrary correlations between any two observations for the same building. β is the coefficient of interest, representing the impact of gas leaks on house sales prices. Under the assumption that there are no other time-variant differences between the treated and control groups (i.e., the “parallel trend assumption”), we are able to obtain a robust causal estimation.

Under the above DID framework, we selected 1,350 houses in the treated group and 188,640 houses in the control group. Houses with nearby gas leaks (within 20 m radius) were dropped from the control group. Thus, all houses in the control group do not have nearby gas leaks. For each treated house, we were able to find about 140 houses in the control group on average. There are 10 grade 1 leaks, 129 grade 2 leaks, and 1,203 grade 3 leaks near the selected treated houses. As of 2019, utilities have reported 31,421 grade 1 gas leaks, 19,345 grade 2 gas leaks, and 30,775 grade 3 leaks in total in MA. In addition, the period of transactions in our analysis ranges from 1987 to 2019. The post-treatment period (houses were traded with nearby gas leaks) starts from 2015 to 2019.

In our study, we use a two-way fixed-effects model, and our estimator is the weighted sum of the average treatment effects in all possible two-group/two-period cells, with weights that might be negative.^32^^,^^33^ To address the issue of potential negative weights, following the approach developed by De Chaisemartin and D'Haultfoeuille,^33^ we calculate the weights attached to our fixed-effects regression and find that 1477 out of 1478 weights are positive. Therefore, our results are unlikely to be affected by negative weights.

Our dataset does not record gas leaks that emerged but were repaired before 2014, and these leaks might cause some of our houses in the pre-treatment period to be in treatment. This impact on our estimation could be minor. Not every building had a nearby gas leak. As of 2019, there were 30,617 grade 2 and grade 3 leaks in total reported to be near home in MA. Those leaks only account for 1.2% of the residential buildings in MA based on 2019 ZTRAX database. The proportion of buildings that were traded with an unobserved gas leak in the pre-treatment period could be very limited.

The sales prices of houses in our repeat-sales model might be different from the prices of other houses because of the problem of “over flipping.” Houses that are frequently transacted may be traded with abnormal (too high or too low) prices compared to the average market level. This concern can be relieved for two reasons. First, most properties in our sample were not traded frequently (e.g., within one or two years). Second, the sales prices of our DID sample and the whole population (all property transactions since 1987) are similar in terms of mean and variance. The average price of DID sample is $462,031 while the average price of all transactions since 1987 is $476,293. The standard deviations of the two groups are 3,176,278 and 2,935,148, respectively.

#### Pre-treatment parallel trend test

To obtain a robust causal estimation under the DID specification, there should be no differential trends between the treated and control groups if the treated group did not receive the treatment. To better support the “parallel trend assumption,” following previous research’s approach,^18^ we conduct a pre-treatment parallel trend test by applying the model,

(Equation 2) $${\ln\;P}_{it}=\sum_{j=1987}^{2017}\beta_{j}T_{i}\cdot Y_{j}+\alpha A_{it}+\phi_{i}+\vartheta_{t}+\tau_{t}+\varepsilon_{it}$$

where $\ln\;P_{it}$ is the log of the sales price of house *i* at time *t.* $T_{i}$ is the treatment group variable and equals 1 if house *i* is in the treatment group and 0 otherwise. $Y_{j}$ is a set of dummy variables indicating years from 1987 to 2017. $A_{it}$ is the building age. $\phi_{i}$ , $\vartheta_{t}$, and $\tau_{t}$ control for individual, yearly, and month-of-year fixed effects, respectively. We only use pre-treatment data in the model. The estimated coefficients for $\beta_{j}$ (j = 1987, …, 2017) are plotted in Figure S1. All the coefficients of $\beta_{j}$ are close to zero and the corresponding 95% confidence intervals include zero. The test results provide supportive evidence that there are no time-variant differences between the treated and control groups in absence of the treatment under our model specification.

### Quantification and statistical analysis

All regression analyses were conducted using Stata 15. Figures 1, 2B, and S3 were generated using QGIS. Figures 2A and S4 were created using Microsoft PowerPoint, while and Figures S1, S2, and S5 were created using Python. All of the statistic details can be found in method details, Tables 1, 2, 3, and 4, and Tables S1 and S2.
