# Supplementary material for: The economic consequences of local gas leaks with evidence from Massachusetts housing market
Source: iScience. 2024 Dec 17;27(12):111483. doi: 10.1016/j.isci.2024.111483 (PMC11700625; doi:10.1016/j.isci.2024.111483)
Supplement: Document S1. Figures S1–S5, Tables S1, S2, Data S1, and S2 [file mmc1.pdf]

iScience, Volume *27*

Supplemental information

The economic consequences of local gas leaks with evidence from Massachusetts housing market

Xingchi Shen, Morgan R. Edwards, Yueming (Lucy) Qiu, and Pengfei Liu

Table S1. Attributes of treated houses in different utility service territories, Related to Table 3

	National Grid Treated houses (n=771)	Columbia Treated houses (n=251)	Eversource Treated houses (n=328)
	(1)	*(2)*	*(3)*
Mean House Characteristics:			
Year Built	1930.24	1934.95	1934.30
Year Remodeled	1982.20	2001.54	1985.58
Lot Size (Acres)	0.28	0.34	0.47
Lot Size (Sq Ft)	12321.18	14633.31	20617.78
No of Stories	2.07	1.79	1.94
Total Rooms	8.05	7.86	8.04
Total Bedrooms	3.64	3.76	3.60
Total Calculated Bath Count	2.29	1.90	1.97
Building Area (SqFt)	2219.14	2047.90	1930.00
Garage (Dummy)	0.31	0.42	0.53
Pool (Dummy)	0.023	0.028	0.027
Land Assessed Value ($)	392469.2	118303.8	175148.2
Fireplace (Dummy)	0.46	0.32	0.36
Surrounding Tree Cover (%)	16.90	15.27	14.04
Mean Census Characteristics			
Median Age	41.11	38.09	39.77
Ratio of High School Diploma	0.14	0.16	0.17
Ratio of Bachelor Degree	0.18	0.12	0.16
Ratio of Master Degree	0.10	0.06	0.08
Ratio of Doctoral Degree	0.025	0.008	0.017
Median Household Income	93177.28	63465.16	70808.22
Personal Income Per Capita	45909.1	29550.12	35040.67
Population density (n/acres)	13.67	9.89	11.33

Note: We obtained the house attributes from ZTRAX database and obtained the demographical attributes from United States Census Bureau's American Community Survey 1-year Estimates, reported at the Census Block Group level, for the release year 2016. The reported mean census attributes in the table are the means of block group level estimates weighted by the number of treated houses in every block group.

Table S2. The impact of surrounding tree covers on home prices, Related to section "Understanding the impact of gas leaks on home prices"

	(1)	(2)
	**2010 tree cover**	**2019 tree cover**
**Outcome: Natural log of home prices (2019$)**		
Tree cover percentage	0.0029***	0.0044***
	(0.0001)	(0.0001)
Building attributes control	Yes	Yes
County-by-year fixed effects	Yes	Yes
Month-of-year fixed effects	Yes	Yes
Adjusted R2	0.4398	0.4393
Observations	186,584	186,470

Note: *** $p < 0.01$, ** $p < 0.05$, * $p < 0.1$. Standard errors are in the parentheses. The outcome is the natural log of home prices that are adjusted to 2019$ for inflation. We restrict the sample to home transactions from 2008 to 2019. In column (1), we use the tree cover data from 2010, and in column (2), we use the tree cover data from 2019. We control for building attributes such as year built, building area, number of stories, rooms, bedrooms, bathrooms, and indicators for the presence of a garage and pool.

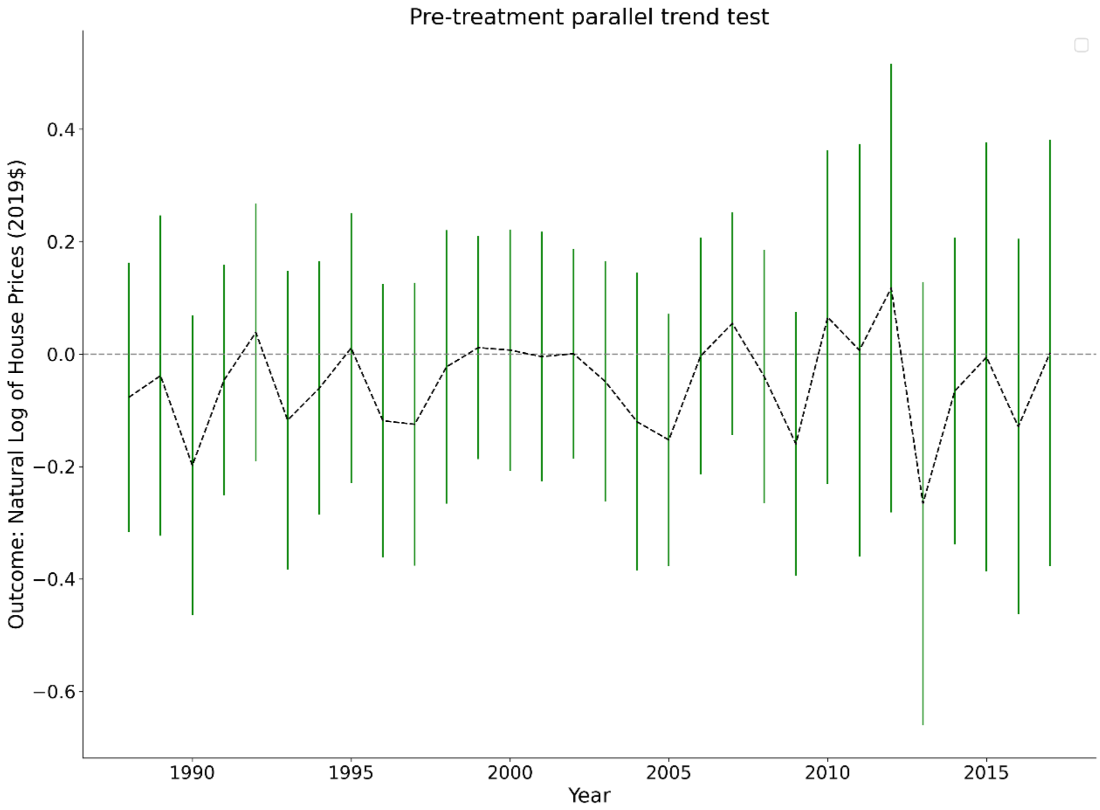

Figure S1. Pre-treatment trend test (dotted line is the point estimators of β_j, green lines are 95% confidence intervals), Related to STAR Methods

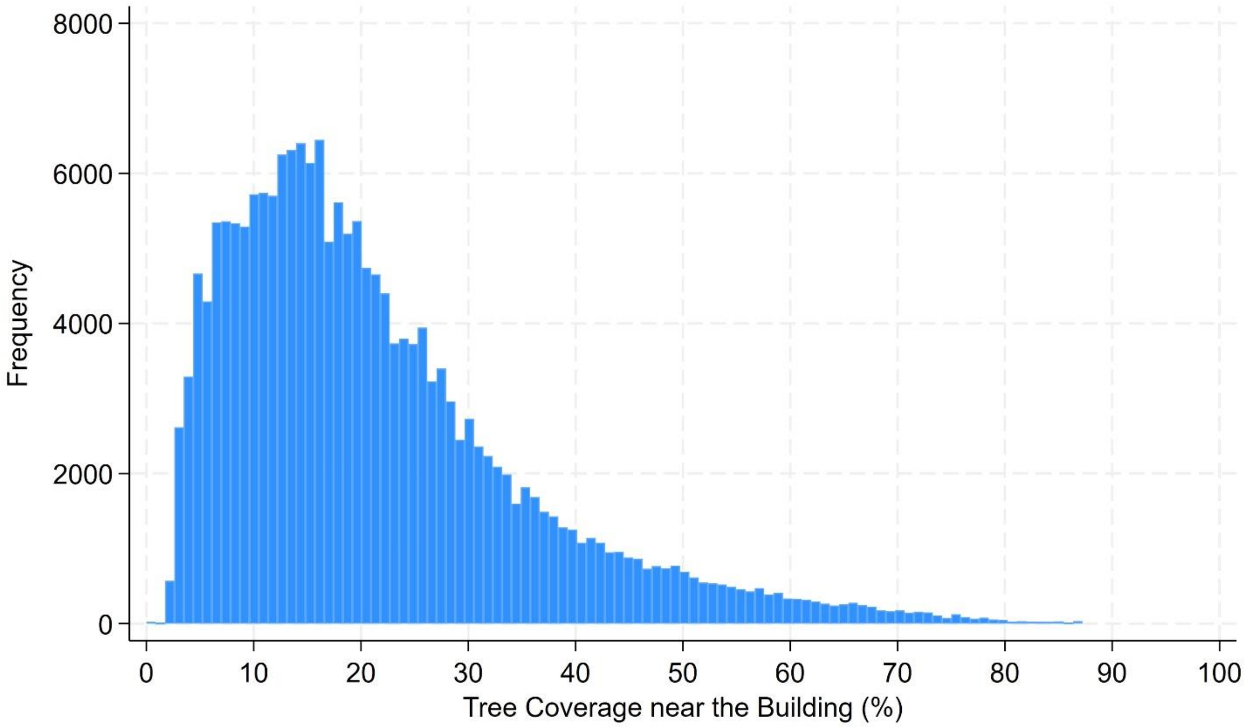

Figure S2. The distribution of tree coverages near houses in the sample, Related to section "Understanding the impact of gas leaks on home prices"

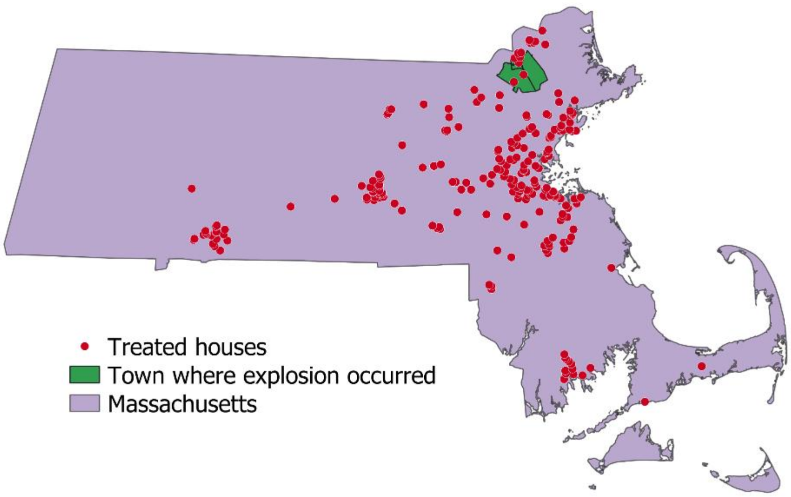

Figure S3. Spatial distribution of treated homes analyzed for explosion information shock, Related to section "Understanding the impact of gas leaks on home prices"

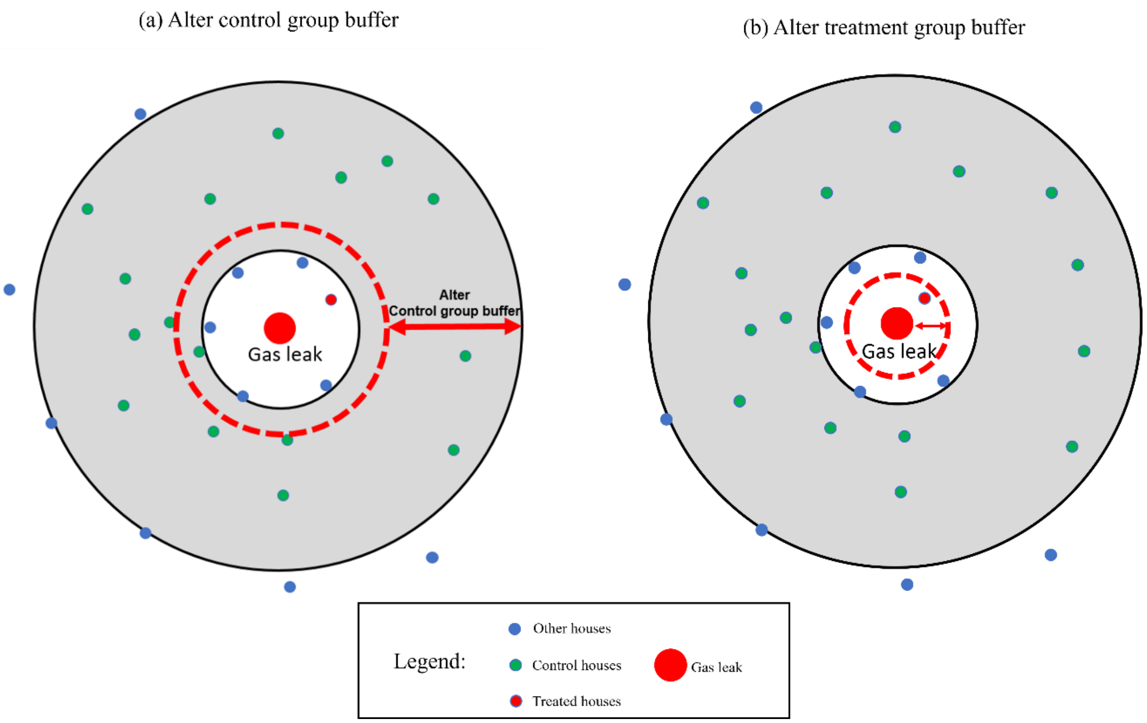

Figure S4. Robustness checks: expanding the internal boundary of the control group buffer and shrinking the outer boundary of the treatment group buffer, Related to STAR methods

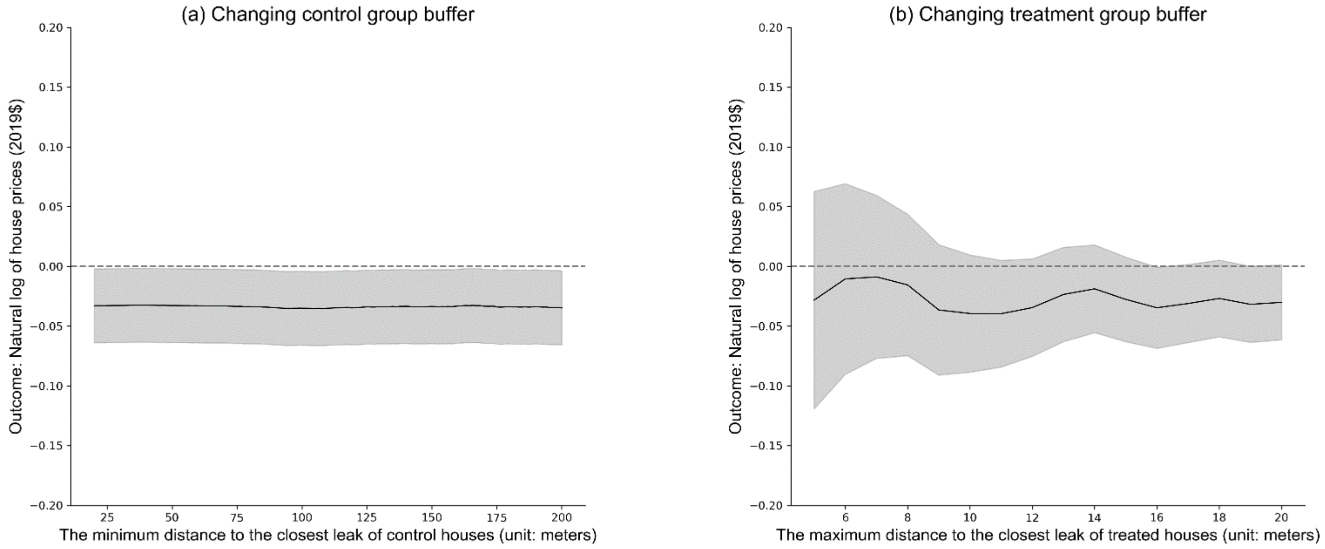

Figure S5. Robustness check results (the black line is point estimators and the shaded area is 95% confidence intervals), Related to STAR methods

Data S1. Robustness check on group buffer length, Related to STAR Methods

In our baseline analysis, we set the radius of treatment group buffer as 20 meters and set the radius of control group buffer as 500 meters. To relive the concerns about choosing group buffer lengths, we conduct additional robustness checks by altering the boundaries of the treatment and control groups.

We first test the assumption of control group buffer. If residents or homebuyers can notice the gas leaks or their effects (e.g., damage to greenery) within the control group buffer, the control group can also be impacted by the treatment, which would underestimate the effects of gas leaks on home value. To address this concern, holding treatment group boundary fixed, we expand the internal boundary of the control group buffer (see subplot (a) in **figure S4**) and re-estimate the treatment effects using the MA sample with treatments of grade 2 & 3 leaks and applying the same DID model (see equation (1)). We change the distance from the internal boundary of the control group to the closest leak from 20 meters to 200 meters continuously. Subplot (a) in **figure S5** presents the robustness check results. We find that our estimations are robust to changing the boundary of the control group. This suggests that gas leaks do not impact home prices outside a radius of 20 meters.

We then conduct a sensitivity analysis by altering the radius of the treatment group buffer (see subplot (b) in **figure S4**). As we choose different radiuses, different houses will be included in the treatment group and our estimated treatment effect could change. In this test, holding the control group boundary fixed, we shrink the radius of treatment group from 20 meters to 5 meters continuously and re-estimate the effects using the same method as above. Subplot (b) in **figure S5** shows the sensitivity analysis results. The treatment effects are consistently negative at a 10% level of statistical significance when the treatment group buffer radius is between 20 meters and 10 meters. When the radius is less than 10 meters, treatment effects are still negative but not statistically significant, which could be due to the small sample size of treated houses. We do not restrict the radius to values below 5 meters since it would lead to very few observations of treated houses. This result shows that our estimations are robust to changing treatment group buffer.

Data S2. The impact of surrounding tree covers on home prices, Related to section "Understanding the impact of gas leaks on home prices"

We provide additional empirical evidence that diminished greenery reduces home prices. To examine how homeowners and homebuyers consider greenery when purchasing a home, we employ a hedonic pricing model to estimate the impact of local greenery on home prices. This approach is a widely used method for assessing individuals' preferences for surrounding amenities in their home purchase decisions. For robustness, we utilized two sources of tree cover data. The primary dataset, from the U.S. Department of Agriculture Forest Service and used in the main text, is from 2019. The secondary dataset is the 2010 Global Tree Cover data, sourced from the Global Land Analysis and Discovery (GLAD) laboratory at the University of Maryland. Both datasets are derived from satellite images and have a resolution of 30m by 30m. Since our tree cover data is in 2010 and 2019, we restrict our sample to transactions after 2008. In our analysis, we regress the log of home sales prices on the percentage of surrounding tree cover, controlling for building attributes such as year built, building area, number of stories, rooms, bedrooms, bathrooms, and indicators for the presence of a garage and pool. We also include county-by-year fixed effects and month-of-year fixed effects. These fixed effects help control for macro trends in housing market conditions within each county and seasonal cycles. The results, using tree cover data from 2010 and 2019 for robustness, are shown in the **table S2**. We find that surrounding tree cover can significantly increase home prices. A one standard deviation increase in tree cover can raise home prices by 5.76%-6.05%. These results strongly support the idea that homebuyers consider surrounding greenery when purchasing a home. Trees offer multiple benefits, such as enhancing views, providing shade, and increasing privacy. Other studies have also found similar results, indicating that homebuyers prefer residential units with higher street-visible greenery [S1].

References

1. Zhang, Y., & Dong, R. (2018). Impacts of street-visible greenery on housing prices: Evidence from a hedonic price model and a massive street view image dataset in Beijing. ISPRS International Journal of Geo-Information, 7(3), 104.
